# Risk and protective factors in gifted children with dyslexia

**DOI:** 10.1007/s11881-015-0106-y

**Published:** 2015-08-13

**Authors:** Sietske van Viersen, Elise H. de Bree, Evelyn H. Kroesbergen, Esther M. Slot, Peter F. de Jong

**Affiliations:** Research Institute of Child Development and Education (RICDE), University of Amsterdam, PO Box 15780, 1001 NG Amsterdam, The Netherlands; Department of Pedagogical and Educational Sciences, Utrecht University, PO Box 80140, 3508 TC Utrecht, The Netherlands

**Keywords:** Bayesian statistics, Case series, Dyslexia, Giftedness, Literacy, Risk/protective factors

## Abstract

This study investigated risk and protective factors associated with dyslexia and literacy development, both at the group and individual level, to gain more insight in underlying cognitive profiles and possibilities for compensation in high-IQ children. A sample of 73 Dutch primary school children included a dyslexic group, a gifted-dyslexic group, and a borderline-dyslexic group (i.e., gifted children with relative literacy problems). Children were assessed on literacy, phonology, language, and working memory. Competing hypotheses were formulated, comparing the core-deficit view to the twice-exceptionality view on compensation with giftedness-related strengths. The results showed no indication of compensation of dyslexia-related deficits by giftedness-related strengths in gifted children with dyslexia. The higher literacy levels of borderline children compared to gifted children with dyslexia seemed the result of both fewer combinations of risk factors and less severe phonological deficits in this group. There was no evidence for compensation by specific strengths more relevant to literacy development in the borderline group. Accordingly, the findings largely supported the core-deficit view, whereas no evidence for the twice-exceptionality view was found. Besides practical implications, the findings also add to knowledge about the different manifestations of dyslexia and associated underlying cognitive factors at the higher end of the intelligence spectrum.

**Electronic supplementary material:**

The online version of this article (doi:10.1007/s11881-015-0106-y) contains supplementary material, which is available to authorized users.

An abundant body of research focuses on the underlying cognitive deficits associated with dyslexia, the severe and persistent reading and/or spelling difficulties at the word level (Snowling, 2000). Research into these cognitive factors has covered various populations of children with dyslexia, including children of different intelligence levels (e.g., Elbro, 2010; Snowling, 2008; see Vellutino, Fletcher, Snowling, & Scanlon, 2004 for a review). However, high-IQ children (i.e., academic giftedness; Winner, 1997) have remained underresearched in this area of study. Results on averagely intelligent children are generalized to high-IQ populations based on the assumption that literacy and intelligence are only moderately related (e.g., Vellutino, Scanlon, & Lyon, 2000). Yet, gifted children are often believed to have a specific cognitive profile of dyslexia-related weaknesses and giftedness-related strengths, affecting the (clinical) expression of dyslexia (e.g., Brody & Mills, 1997; Foley Nicpon, Allmon, Sieck, & Stinson, 2011). The present study aims to test and extend theories on dyslexia and investigate possibilities for compensation by providing more insight in the underlying cognitive profiles of gifted children through the assessment of risk and protective factors at the group and individual levels.

Current understanding of the etiology of learning disabilities such as dyslexia is best explained by the multiple deficit model (e.g., Bishop & Snowling, 2004; Pennington, 2006; Pennington et al., 2012; Ramus et al., 2003; Wolf & Bowers, 1999). According to this model, learning disabilities arise as a result of the interaction between multiple cognitive risk and protective factors that are influenced by both genes and the environment during development. The model is probabilistic in nature, with risk factors increasing the probability of the emergence of a learning disability and protective factors decreasing this probability. Considering that this model is one of the few to specifically take protective factors into account as part of the development of learning disabilities (Pennington, 2006), the multiple deficit model provides the most suitable framework for investigating the role of protective factors in the development of dyslexia in the current study.

Risk factors associated with dyslexia are generally believed to lie in phonological processing, which is proposed as a primary source of reading disabilities (Vellutino et al., 2004). The most important deficits concern phonological awareness (PA; i.e., detection and manipulation of speech sounds in spoken words), verbal short-term memory (VSTM; i.e., the temporary storage of phonological representations), and rapid automatized naming (RAN; i.e., access to and quick retrieval of orthographic and phonological representations from long-term memory; Boets et al., 2010; de Jong & van der Leij, 2003; Snowling, 2001; Wagner & Torgesen, 1987). The dominant and commonly accepted view regarding these underlying cognitive risk factors is that they are the core deficits of dyslexia and are unaffected by intelligence (hereafter the “core-deficit view”; Stanovich & Siegel, 1994). Children of high and low intelligence are assumed to display no marked differences in the underlying cognitive processes that lie at the basis of their reading disability (Stanovich, 1996). Nonetheless, despite its strong claims that have both theoretical and clinical consequences, the core-deficit view has never been tested in a gifted population.

A contrasting view on the role of intelligence in dyslexia can be found in the literature on twice-exceptionality (i.e., the co-occurrence of giftedness and a learning disability within an individual; Brody & Mills, 1997; see Foley Nicpon et al., 2011 for a review), which specifically addresses the role of protective factors. Protective factors are generally only mentioned in relation to “compensation.” Compensation pertains to the moderation of the effect of a cognitive risk factor by an additional or co-occurring protective factor (e.g., Snowling, Gallagher, & Frith, 2003). According to this “twice-exceptionality” view, the cognitive profile of twice-exceptional children is assumed to consist of high ability-related protective factors (providing possible resources for compensation) and disability-related risk factors (e.g., Assouline, Foley Nicpon, & Whiteman, 2010; Brody & Mills, 1997; Foley Nicpon et al., 2011). The combination of risk and protective factors may result in higher literacy levels than might be expected based on the underlying deficit, which is also referred to as “masking” (e.g., Lovett & Lewandowski, 2006; Silverman, 2003). Accordingly, compensation is attested when, while matched on literacy performance, the cognitive profiles of gifted children with dyslexia are characterized by more severe deficits than averagely intelligent children with dyslexia, as otherwise there would be no room for protective factors to compensate. Protective factors proposed to be involved in compensation of phonological deficits include visual memory and perceptual speed (Snowling, 2001), orthographic knowledge (Stanovich & Siegel, 1994; van der Leij & van Daal, 1999), and/or general language skills (Nation & Snowling, 1998; Snowling, 2001, 2008; Snowling et al., 2003).

So far, only two studies have investigated risk and protective factors in gifted children with dyslexia. Berninger and Abbott (2013) found that *verbally* gifted children with dyslexia outperformed verbally average-performing children with dyslexia on reading, spelling, and language skills, but not on the cognitive deficits associated with dyslexia, phonological and orthographic storage and processing, and RAN. A recent study by van Viersen, Kroesbergen, Slot, and de Bree (2014) on the behavioral and cognitive characteristics of a group of overall gifted children with dyslexia showed that this group exhibited weaknesses in PA and RAN, but not in VSTM, and strengths in verbal and visuospatial working memory (WM) and language skills compared to typically developing (TD) children. Furthermore, their reading and spelling performance was in between that of children with dyslexia and TD children.

The findings of Berninger and Abbott (2013) indeed point toward compensation of underlying deficits by specific strengths, resulting in a certain degree of masking of literacy difficulties compared to averagely intelligent children with dyslexia. Based on their results, more severe cognitive deficits can be expected in gifted children with dyslexia when both groups are matched on literacy performance. The findings by van Viersen et al. (2014) are less easy to interpret, as the underlying deficits were reported to be less severe in gifted children with dyslexia and results were not controlled for literacy level. This is necessary for assessing real differences between cognitive profiles of both dyslexic groups and making direct claims about compensation or masking. Accordingly, more research into the cognitive profiles of gifted children with dyslexia, and specifically the combination between risk and protective factors, is needed.

An even more interesting group of gifted children to investigate in the context of risk and protective factors and compensation and masking concerns gifted children who read poorly compared to their IQ, but do not meet standards of low achievement needed for a diagnosis (i.e., “borderline-dyslexic” children). These children are often not eligible for treatment. In other words, these borderline children show *relative* literacy problems, while their intelligence levels, and therefore general “compensatory resources,” are equal to those of gifted children that do reach the diagnostic threshold for dyslexia. Charting the cognitive risk and protective factors of both groups (and individual profiles within both groups) provides important insights in possibilities for compensation and masking and explains their differences in literacy performance, thereby putting both the core-deficit and twice-exceptionality views to the test.

In the present study, we aimed to test and extend theories on dyslexia and possibilities for compensation by investigating profiles of underlying cognitive risk and protective factors of two populations of gifted children, both at the group and individual levels. First, the cognitive profiles of gifted children with dyslexia and averagely intelligent children with dyslexia were compared controlling for differences in literacy levels, by reanalyzing the data of two subgroups presented in van Viersen et al. (2014). Based on the core-deficit view, it was hypothesized that gifted children with dyslexia and children with dyslexia would show equally large underlying deficits when controlling for literacy, with protective factors being present but independent of the risk profile. In contrast, the concomitant occurrence of larger deficits and more pronounced strengths in gifted children with dyslexia would support the twice-exceptionality view on compensation. Second, the cognitive profiles of gifted children with dyslexia and borderline children were compared controlling for differences in overall intelligence. Given the higher literacy level in the borderline group, it was hypothesized that this difference could be explained by either smaller deficits (core-deficit view) or the presence of *specific* protective factors more relevant for compensation, such as language skills (twice-exceptionality view), or both. An individualized approach toward both gifted groups was required for providing more information on *combinations* of deficits within children. It was hypothesized that the groups would not differ in the numbers of risk and protective factors overall. Instead, the breadth/depth of underlying deficits (core-deficit view) and/or specific strengths (twice-exceptionality view) may account for differences between both groups in literacy development.

## Methods

### Participants

A total of 73 Dutch primary school children (56.2 % boys) from grades 2 to 4 participated in the study. The children were recruited through contacts with school teachers or school psychologists and calls on the websites of educational magazines and clinical institutions. Informed consent was obtained from all participants and their parents. The inclusion criterion for giftedness was set at a full IQ score >125 or a 95 % reliability interval tapping at least 130 in the case of a short form. In addition, the criteria for dyslexia were set at (1) a significant discrepancy between IQ and reading *or* spelling performance of at least 2 SD (Snowling, 1998) and (2) below average scores on reading *or* spelling (lowest 10–15 % or a standard score ≤6). The latter criterion is in line with an official diagnosis of dyslexia in The Netherlands (Kleijnen et al., 2008) and was the specific criterion that had to be fulfilled by the averagely intelligent children with dyslexia. The gifted children were divided in a group of children that met both criteria (i.e., gifted children with dyslexia; *n* = 26, 65.4 % boys) and a borderline-dyslexic group (*n* = 14, 57.1 % boys) that only met the first criterion. The children in the borderline group showed relative literacy problems compared to their high IQ but did not reach the diagnostic cutoff for a dyslexia diagnosis.

Table 1 displays the division of age, intelligence, and sex in the groups and mean literacy levels. The groups significantly differ in age (*F*[2, 70] = 9.52, *p* < .001); the borderline group is significantly younger than both other groups (*p* < .01). As expected, the groups also differ significantly in intelligence (*F*[2, 70] = 126.76, *p* < .001). The gifted groups have about equal intelligence levels (*p* = .37), which differ significantly from the averagely intelligent dyslexic group (*p* < .001). All three groups significantly differ in word reading (*F*[2, 70] = 26.83, *p* < .001) and nonword reading levels (*F*[2, 70] = 24.91, *p* < .001), following the pattern borderline > gifted/dyslexic > dyslexic. There was a main effect of spelling level (*F*[2, 70] = 10.26, *p* < .001); the dyslexic group showed significantly lower scores than both gifted groups (*p* < .01).

**Table 1:** Sample size and means and standard deviations for age, IQ score, and literacy measures per group

Variable	Gifted + dyslexia^a^ (*n* = 26)		Borderline (*n* = 14)		Dyslexia^a^ (*n* = 33)	
	*M*	SD	*M*	SD	*M*	SD
Age (months)	108.77	8.14	99.00	10.49	113.85	12.39
IQ (total)	132.50	8.05	135.29	4.58	98.70	11.41
Word reading^b^	5.77	2.75	9.64	1.69	4.06	2.33
Nonword reading^c^	6.27	2.20	9.14	1.61	4.64	2.00
Spelling^d^	3.12	12.08	10.56	13.54	−6.93	13.19

^a^Data reported in van Viersen et al. (2014)

^b^Eén Minuut Test (EMT; Brus & Voeten, 1999), maximum score = 116

^c^Klepel (van den Bos, lutje Spelberg, Scheepstra, & de Vries, 1994), maximum score = 116

^d^PI-dictee (Geelhoed & Reitsma, 2000), instruction-residual

## Instruments

### Intelligence

A short form of the *Wechsler Intelligence Scale for Children NL, Third edition* (WISC-III; Kort et al., 2005) was used to estimate the general cognitive abilities of the children, consisting of the performance subtests picture completion and block design and the verbal subtests similarities and vocabulary. Full IQ scores (*M* = 100, SD = 15) were computed based on the formula provided by Kaufman, Kaufman, Balgopal, and McLean (1996) and used for inclusion. The reported reliability and validity quotients of this short form are all greater than .83 (Kaufman et al., 1996). Children were not re-assessed, and recent results were used if a complete version of the WISC-III had already been administered in the past 2 years.

### Literacy

Timed word reading and decoding speed of nonwords were measured using *Eén Minuut Test* (EMT; Brus & Voeten, 1999) and *Klepel* (van den Bos et al., 1994), respectively. The child had 1 (EMT) and 2 min (Klepel) to accurately read as many (non)words as possible. In both tests, word length increased from one to four syllables. Raw scores were the number of correctly read words or nonwords, with a maximum of 116 words, which were transformed into age-referenced norm scores (*M* = 10, SD = 3) for the inclusion. Internal consistency is .90 for EMT and .92 for Klepel (Evers et al., 2009–2012).

A short form of the *PI-dictee* (Geelhoed & Reitsma, 2000) was used to measure spelling dictation at word level. The short form contained eight sets of seven words, with each set representing specific spelling categories. The test was discontinued when the child made six or more errors in one set. Raw scores are the total number of correctly written words. For the analyses, the total raw score was transformed into an instruction-residual after partialling out the number of months of instruction in spelling (i.e., computed by 10 months of instruction per year of education). Internal consistency of the full version varies between .90 and .93 (Evers et al., 2009–2012).

### Phonology

PA was assessed using the *Fonemische Analyse Test* (FAT; van den Bos, lutje Spelberg, & de Groot, 2011), which measured the ability to quickly analyze and manipulate phonemes in words in two subtests with 12 items each. The first subtest targets phoneme deletion (e.g., *kraal* “bead” without /k/ is *raal*) and the second subtest is a spoonerism task (e.g., transposing onset phonemes of *Kees Bos* to *Bees Kos*). For both tasks, raw response time in seconds was transformed into an age-referenced norm score (*M* = 8–12). In addition, the number of correct answers per minute was computed based on raw speed and accuracy scores per task and used for the analyses. Internal consistency of the test is .93 (Evers et al., 2009–2012).

The task *Continu Benoemen & Woorden Lezen* (CB&WL; van den Bos & Lutje Spelberg, 2007) was used to assess RAN. The test consisted of four subtests (colors, digits, pictures, and letters) measuring the child’s naming speed. The child is asked to correctly name 50 items (5 types per subtest, e.g., red, yellow, blue, green, and black) as quickly as possible. Raw scores (i.e., naming time in seconds) of the subtests could be transformed into age-referenced norm scores (*M* = 8–12). The raw scores of the letters and digits subtests were combined into a (mean) alphanumeric RAN variable, and the colors and pictures subtests were combined into a (mean) non-alphanumeric RAN variable for the analyses. Internal consistency of the test varies between .79 and .87 (Evers et al., 2009–2012).

The subtest digit recall of the *Automated Working Memory Assessment* battery (AWMA; Alloway, 2007) was used to measure VSTM, which required the child to recall several series of digits of increasing length. All subtests of this instrument were discontinued after three incorrect answers. Raw accuracy scores were used in the analysis and could also be transformed into age-referenced norm scores (*M* = 85–115). Test-retest reliability is .89 (Alloway, Gathercole, Kirkwood, & Elliot, 2009).

### Working memory

The verbal and visuospatial short-term and WM components were measured using subtests of the AWMA battery (Alloway, 2007). Backward digit recall, in which the child recalled increasing series of digits backwards, was used to measure verbal working memory (VWM). Visuospatial working memory (VSWM) was measured by the subtests spatial span (SS), in which the child had to evaluate figures by mental rotation, classify them as “the same” or “opposite,” and recall the position of a red dot in an empty figure, and the subtest odd-one-out (OO), in which the child had to indicate the odd figure out of increasingly complex sequences of three figures and recall the odd figure in a matrix. Raw scores on both subtests were combined into a mean VSWM variable. Visuospatial short-term memory (VSSTM) was measured using the subtest dot matrix, in which the child had to recall the position of increasingly complex series of red dots in an empty matrix. Raw accuracy scores were used in the analyses but could also be transformed into age-referenced norm scores (*M* = 85–115). Test-retest reliabilities were .86, .79, .88, and .85, respectively (Alloway et al., 2009).

### Language

The *Clinical Evaluation of Language Fundamentals-4 NL* (CELF; Kort, Schittekatte, & Compaan, 2010) was used to measure grammar and vocabulary. The subtest formulated sentences was used to assess the child’s grammar skills. Here, the child had to formulate sentences (FS) about actions or situations that were expressed in pictures using a targeted word or phrase. The subtest word classes 2 (WC2), in which the child chose two related words and described their relationship, measured vocabulary. Raw accuracy scores (FS max = 40, WC2 max = 40) were used in the analyses and could also be transformed into age-referenced norm scores (*M* = 8–12). Internal consistency of the subtests is .78 and .87, respectively (Evers et al., 2009–2012).

### Procedure

The assessments were performed by supervised graduate students, who all received an extensive training in using the test battery described above. Children were tested either in a clinic, at school, or in their homes within one 2–3-h session, with plenty of breaks in between tests. The duration of the assessment depended on the availability of recent and relevant test results on the instruments included in the test battery (i.e., IQ not older than 2 years, literacy not older than 6 months). The test results were summarized in a short report, evaluated by a licensed school psychologist, and reported back to the parents of the participating children. Any diagnostic uncertainties were resolved during joint evaluation meetings.

### Analyses

#### Group analyses

To test the first two hypotheses concerning the group comparisons between the gifted children with dyslexia and the averagely intelligent children with dyslexia (first hypothesis) and between the gifted children with dyslexia and the borderline children (second hypothesis), Bayesian statistics were used instead of traditional frequentist statistics (i.e., here, the frequentist alternative would be MANCOVA). Bayesian statistics provide important benefits over traditional methods for the current study. First, in contrast to null hypothesis testing, Bayesian model selection offers the possibility to formulate and evaluate informative hypotheses based on prior knowledge by using equality and inequality constraints between groups and compare competing hypotheses that represent specific expectations or views (Klugkist, Laudy, & Hoijtink, 2005). Second, the outcome of the analyses is a Bayes factor (BF), which represents the amount of evidence in favor of one hypothesis compared to another (Kass & Raftery, 1995), instead of a traditional *p* value. As such, Bayesian model selection offers solutions to analytical problems related to multiple testing, such as alpha inflation and loss of power after correcting the alpha level (Klugkist, van Wesel, & Bullens, 2011). Third, Bayesian analyses are not based on normality or asymptotic assumptions, making it a useful alternative when working with small sample sizes (Gill, 2008) or samples drawn from one side of the distribution (e.g., children with disabilities). Finally, the obtained parameter estimates (i.e., posterior means and posterior standard deviations) include the effect of the covariates and can directly be used to make inferences about group differences. The analyses were conducted with the BIEMS software package (Mulder, Hoijtink, & Klugkist, 2010; Mulder, Hoijtink, & de Leeuw, 2012; Mulder et al., 2009).

For the analyses, each hypothesis was translated into a statistical hypothesis with (in)equality constrained parameters (Klugkist et al., 2005). In this study, the parameters were the group means (i.e., μ) on the cognitive risk and protective factors. The statistical hypotheses are displayed and further explained in Table 2. First, each of the informative hypotheses is compared to the alternative hypothesis by calculating the BF_i,u_ values. A BF larger than 1 indicates support in favor of the informative hypothesis, whereas a BF smaller than 1 provides evidence in favor of the alternative hypothesis. A rule of thumb for the interpretation of the BF is provided by Kass and Raftery (1995), stating that a BF between 1 and 3 represents a small effect, a BF between 3 and 10 can be considered as substantial evidence supporting the informative hypothesis under investigation, and a BF above 10 indicates a large effect.

**Table 2:** Investigated models, statistical hypotheses, and translation for Bayesian analyses

Model	Statistical hypothesis	Translation
Gifted + dyslexia vs. dyslexia		
Weaknesses		
Model 0	μ_D_, μ_GD_	Alternative hypothesis or unconstrained model
Model 1	μ_D_ > μ_GD_	Twice-exceptionality view: Dyslexic children have higher scores on the cognitive risk factors than gifted/dyslexic children, indicating more severe deficits in the gifted/dyslexic group
Model 2	μ_D_ = μ_GD_	Core-deficit view: Both groups have about equal scores on the cognitive risk factors, indicating that the groups are comparable
Strengths		
Model 0	μ_D_, μ_GD_	Alternative hypothesis or unconstrained model
Model 1	μ_D_ < μ_GD_	Twice-exceptionality view: Dyslexic children have lower scores on the cognitive protective factors than gifted/dyslexic children, indicating more pronounced strengths in the gifted/dyslexic group
Model 2	μ_D_ = μ_GD_	Both groups have about equal scores on the cognitive protective factors, indicating that the groups are comparable
Gifted + dyslexia vs. borderline		
Weaknesses		
Model 0	μ_B_, μ_GD_	Alternative hypothesis or unconstrained model
Model 1	μ_B_ > μ_GD_	Core-deficit view: Borderline children have higher scores on the cognitive risk factors than the gifted/dyslexic children, indicating less severe deficits in the borderline group
Model 2	μ_B_ = μ_GD_	Both groups have about equal scores on the cognitive risk factors, indicating that the groups are comparable
Strengths		
Model 0	μ_B_, μ_GD_	Alternative hypothesis or unconstrained model
Model 1	μ_B_ > μ_GD_	Twice-exceptionality view: Borderline children have higher scores on the cognitive protective factors than the gifted/dyslexic children, indicating more pronounced strengths in the borderline group
Model 2	μ_B_ = μ_GD_	Both groups have about equal scores on the cognitive protective factors, indicating that the groups are comparable

If one or more of the informative hypotheses received more support from the data than the alternative hypothesis, these competing hypotheses can be compared by calculating the posterior model probability (PMP) for each model (assuming prior probabilities to be equal for all models). This is done by dividing the BF_i,u_ of each model by the sum of BFs of the other relevant models. The PMP represents the relative support for a specific hypothesis within a set of hypotheses (Klugkist et al., 2011). Note that in a Bayesian framework, the term “probability” refers to a degree of belief and relates to the probability that a hypothesis is true (Klugkist et al., 2011). When the difference between the PMPs of two models is smaller than .05, the models are considered to be equally supported by the data. The parameter estimates were used to make more detailed inferences about differences between the groups. Group differences on cognitive risk and protective factors were evaluated using both multivariate and univariate testing.

To adjust for differences in literacy levels between the gifted children with dyslexia and the averagely intelligent children with dyslexia, age and a literacy composite were used as covariates. The literacy composite consisted of the average *z*-scores of the word reading and nonword reading norm scores and the instruction-residual of the raw spelling score. Age and IQ score were used as covariates for the comparison of the cognitive profiles between the gifted children with dyslexia and the borderline children.

#### Individual analyses

A case series analysis was conducted on the data of the gifted children with dyslexia and the borderline children for assessing individual differences between cognitive profiles of strengths and weaknesses of these children (third hypothesis). For each cognitive risk factor, impaired performance was defined as a standard score on the relevant measures below a cutoff of −1 standard deviation relative to the norm-based mean. This criterion is also used in the official diagnosis of dyslexia in The Netherlands (SDN, 2008) and has been found useful in previous research on subgroups of poor readers (e.g., Catts, Hogan, & Fey, 2003; Ramus et al., 2003; Nag & Snowling, 2011). A cutoff of +1 standard deviation relative to the norm-based mean was used to identify cognitive protective factors. In addition, risk and protective factors were combined into strengths and weaknesses, representing dyslexia-related and giftedness-related cognitive domains. Below average performance on either or both the deletion and spoonerism task was counted as a weakness in PA. Similarly, impaired performance on either or both alphanumeric and non-alphanumeric RAN was considered a weakness in RAN. As VSTM has been found to be a potential strength in gifted children with dyslexia (van Viersen et al., 2014), it could be mapped as either a risk or a protective factor per child. Above average performance on either or both VSTM and VWM was considered a strength in verbal WM abilities, and high performance on either or both VSSTM and VSWM was counted as a strength in visual WM skills. High scores on either or both grammar and vocabulary were considered a strength in language skills.

## Results

### Group comparisons

For the first part of the analyses, cognitive profiles of gifted children with dyslexia and averagely intelligent children with dyslexia were compared, controlling for differences in literacy level. Table 3 shows the posterior means and standard deviations of the gifted children with dyslexia and the children with dyslexia and the BFs and PMPs for the three models in the analyses of the cognitive factors.

**Table 3:** Posterior means (PM) and standard deviations (PSD) adjusted for age and literacy performance and Bayes factors (BF) and posterior model probabilities (PMP) of the three models for the cognitive risk and protective factors of the gifted/dyslexic and dyslexic children

Skill/component	Dyslexia (*n* = 33)		Gifted + dyslexia (*n* = 26)		Model 0 (μ_D_, μ_GD_)		Model 1		Model 2 (μ_D_ = μ_GD_)	
	PM	PSD	PM	PSD	BF	PMP	BF	PMP	BF	PMP
Risk factors							(μ_D_ > μ_GD_)^a^			
Multivariate					*1.00*	.84	0.00	.00	0.19	.16
Univariate										
Phoneme deletion	11.32	1.34	17.51	1.68	*1.00*	.98	0.00	.00	0.02	.02
Phoneme transposition	1.90	0.07	3.08	0.09	*1.00*	.96	0.00	.00	0.03	.03
RAN alphanumeric	33.71	1.09	32.84	1.38	1.00	.28	0.58	.16	*2.00*	.56
RAN non-alphanumeric	55.68	3.01	53.33	3.72	1.00	.35	0.36	.12	*1.53*	.53
VSTM	22.63	0.62	25.98	0.77	*1.00*	.94	0.01	.01	0.05	.05
Protective factors							(μ_D_ < μ_GD_)			
Multivariate					1.00	.06	*16.1*	.94	0.03	0.00
Univariate										
Verbal WM	10.40	0.47	12.76	0.58	1.00	.32	*1.98*	.63	0.17	.06
Visuospatial STM	21.34	0.61	26.10	0.77	1.00	.33	*2.00*	.67	0.00	.00
Visuospatial WM	14.93	0.64	19.99	0.81	1.00	.33	*2.00*	.67	0.00	.00
Grammar	26.32	0.83	27.50	1.04	1.00	.24	*1.62*	.39	*1.52*	.37
Vocabulary	13.50	0.67	17.70	0.84	1.00	.33	*2.00*	.66	0.01	.00

Italicized values indicate BFs of models that received most support from the data

^a^μ_1_ < μ_2_ for both RAN variables, because higher numbers indicate slower performance

The multivariate results show that overall the alternative hypothesis received the most support from the data for the *risk* factors (model 0; PMP = .84). The univariate results show that this is the case for both PA tasks and VSTM (PMPs .98–.94). The posterior means of the unconstrained model indicate that the gifted children with dyslexia had neither equal nor lower scores on PA and VSTM compared to the children with dyslexia, but instead had higher scores. This indicates that gifted children with dyslexia may have *less* severe deficits in these areas. For RAN, model 2 received the most support from the data, about two times more than the alternative hypothesis, with the probabilities that the hypothesis under model 2 is true, varying between .56 and .53. The posterior means show that controlling for literacy level, the gifted children with dyslexia and the children with dyslexia have similar scores on RAN.

The multivariate results for the *protective* factors show that overall model 1 received the most support from the data, about 16 times more than the alternative hypothesis, indicating a large effect (PMP = .94). This is supported by univariate results, showing that indeed model 1 received the most support from the data for all protective factors (PMPs .67–.63), except for grammar where the difference between both models is too small to interpret (ΔPMP < .05). The posterior means show that on verbal WM, visuospatial STM and WM, and vocabulary, the gifted children with dyslexia obtained higher scores than the children with dyslexia, whereas on grammar both groups had comparable scores when controlled for differences in literacy levels. Overall, the results show that the gifted children with dyslexia have equal or even less severe deficits on risk factors than averagely intelligent children with dyslexia, and indeed strengths on a broad range of protective factors.

For the second part of the analyses, gifted children with dyslexia and borderline children were compared on their cognitive profiles, controlling for differences in overall intelligence. Table 4 displays the posterior means and standard deviations of the gifted children with dyslexia and the borderline children and the BFs and PMPs for the three models in the analyses of the risk and protective factors.

**Table 4:** Posterior means (PM) and standard deviations (PSD) adjusted for age and IQ and Bayes factors (BF) and posterior model probabilities (PMP) of the three models for the cognitive risk and protective factors of the gifted/dyslexic and borderline children

Skill/component	Gifted + dyslexia (*n* = 26)		Borderline (*n* = 14)		Model 0 (μ_B_, μ_GD_)		Model 1 (μ_B_ > μ_GD_)^a^		Model 2 (μ_B_ = μ_GD_)	
	PM	PSD	PM	PSD	BF	PMP	BF	PMP	BF	PMP
Risk factors										
Multivariate					1.00	.14	2.89	.40	*3.28*	.46
Univariate										
Phoneme deletion	17.94	2.21	20.19	3.98	1.00	.26	*1.64*	.42	1.28	.33
Phoneme transposition	3.10	0.16	3.77	0.28	1.00	.27	*1.69*	.45	1.06	.28
RAN alphanumeric	34.01	2.51	32.32	4.50	1.00	.25	*1.50*	.38	*1.48*	.37
RAN non-alphanumeric	55.98	4.62	53.36	8.12	1.00	.25	*1.55*	.39	*1.38*	.35
VSTM	25.91	0.56	25.45	0.98	1.00	.30	0.69	.21	*1.59*	.48
Protective factors										
Multivariate					1.00	.16	1.36	.22	*3.76*	.61
Univariate										
Verbal WM	12.55	0.34	13.31	0.60	1.00	.26	*1.58*	.41	1.24	.33
Visuospatial STM	25.18	0.72	25.52	1.29	1.00	.25	1.19	.30	*1.83*	.46
Visuospatial WM	19.31	0.58	20.03	1.04	1.00	.25	*1.44*	.36	*1.55*	.39
Grammar	26.56	0.92	25.76	1.66	1.00	.31	0.62	.19	*1.65*	.50
Vocabulary	18.29	0.76	18.03	1.32	1.00	.27	0.86	.24	*1.78*	.49

Italicized values indicate BFs of models that received most support from the data

^a^μ_B_ < μ_GD_ for both RAN variables, because higher numbers indicate worse performance

For the *risk* factors, the multivariate results show that overall model 2 received the most support from the data, about three times more than that of the alternative hypothesis, indicating a small to medium effect (PMP = .46). Yet, the univariate results show that model 1 received the most support from the data for the PA tasks (PMPs .42–.45). The posterior means indicate that the borderline children indeed have higher scores on both PA tasks than the gifted children with dyslexia, indicating less severe deficits. For RAN, models 1 and 2 received about equal support from the data (ΔPMP < .05), although the posterior means show that the gifted children with dyslexia were somewhat slower on the tasks than the borderline children. In other words, both groups have comparable RAN levels, but the borderline group has significantly better literacy skills. This indicates that the children in the borderline group have *relatively* larger RAN deficits than the gifted children with dyslexia. For VSTM, model 2 received the most support from the data (PMP = .48), indicating that both groups obtained comparable scores.

For the *protective* factors, the multivariate results show that overall model 2 received the most support from the data, about four times more than that of the alternative hypothesis, indicating a medium effect (PMP = .61). The univariate results show that indeed model 2 received the most support for the visuospatial STM and language factors (PMPs .50–.39), but not for verbal WM, for which, as for the risk factors, model 1 received the most support from the data (PMP = .41). For visuospatial WM, models 1 and 2 received about equal support from the data (ΔPMP < .05). The posterior means show that the gifted children with dyslexia have lower scores on verbal WM than the borderline children. On the other protective factors, both groups appear to have comparable scores, showing that borderline children have neither more pronounced strengths in general nor more pronounced strengths in, for example, language skills that are considered more relevant to literacy development. Overall, the results show that borderline children have (at most) equal or less severe deficits on risk factors than gifted children with dyslexia, and a comparable and about equally strong set of protective factors, apart from verbal WM.

### Case series

Finally, a case series analysis was performed on the gifted children with dyslexia (Table 5) and the borderline children (Table 6).^1^ Instead of showing differences in group averages on risk and protective factors (size/severity), this case series provides more information about heterogeneity within both gifted groups and individual differences in numbers of risk and protective factors and breadth/depth of strengths and weaknesses.

**Table 5:** Findings from the case series showing the cognitive risk and protective factors observed among gifted/dyslexic children with literacy impairments

Case no.	Risk factors					Protective factors					IQ score	Literacy composite	Number of weaknesses	Number of strengths
	PA deletion SS < 8	PA spoonerism SS < 8	RAN alphanumeric SS < 8	RAN non-alphanumeric SS < 8	VSTM SS < 85 SS > 115	VWM SS > 115	VSSTM SS > 115	VSWM SS > 115	Grammar SS > 12	Vocabulary SS > 12				
208	−		−	−	+		+	+	+		133	−0.53	2	3
6			−	−			+		+		>145	−0.41	1	2
30		−	−	−			+	+	+	+	138	−1.08	2	2
197	−		−	−	+	+	+	+			133	−0.65	2	2
22				−				+	+	+	126	−1.19	1	2
124	−	−	−	−			+				133	−0.64	2	1
29	−	−	−	−			+	+			124	−0.57	2	1
83	−		−	−	+		+				123	−1.03	2	2
93			−	−			+				124	−1.00	1	1
87					+	+	+	+	+	+	>145	0.10	0	3
49							+				130	−0.15	0	1
91	−					+	+	+		+	133	−0.21	1	3
35			−				+	+			137	0.19	1	1
67	−	−						+	+		125	−0.20	1	2
109		−	−	−			+		+		>145	−0.08	2	2
1			−	−			+	+			129	−0.56	1	1
51			−	−			+				121	−0.19	1	1
15	−	−	−				+				123	−0.94	2	1
204				−			+		+	+	139	0.80	1	2
60				−							137	0.05	1	0
53	−	−		−	−		+	+		+	145	0.19	3	2
193			−	−		+	+	+	+		142	−0.11	1	3
125	−		−		−		+	+			135	0.49	3	1
65			−				+	+			121	−0.09	1	1
13							+		+	+	124	−0.05	0	2
34									+		135	0.47	0	1
Total	10	7	16	16	2/4	4	22	14	11	7	132.5	−0.29	1.31	1.65

*PA* phonological awareness, *RAN* rapid automatized naming, *VSTM* verbal short-term memory, *VWM* verbal working memory, *VSSTM* visuospatial short-term memory, *VSWM* visuospatial working memory, *SS* standard score

**Table 6:** Findings from the case series showing the cognitive risk and protective factors observed among the borderline cases with relative literacy problems

Case no.	Risk factors					Protective factors					IQ score	Literacy composite	Number of weaknesses	Number of strengths
	PA deletion SS < 8	PA spoonerism SS < 8	RAN alphanumeric SS < 8	RAN non-alphanumeric SS < 8	VSTM SS < 85 SS > 115	VWM SS > 115	VSSTM SS > 115	VSWM SS > 115	Grammar SS > 12	Vocabulary SS > 12				
7			−		−	+	+	+	+	+	132	1.04	2	3
5	−	−					+	+		+	138	0.86	1	2
130		−	−	−		+					135	−0.21	2	1
131	−	−				+		+			137	0.70	1	2
69				−					+		135	0.55	1	1
14	−		−	−	−			+		+	136	−0.32	3	2
92		−									130	0.68	1	0
31			−	−			+	+		+	137	−0.17	1	2
185			−	−							130	0.25	1	0
132			−	−				+		+	131	0.37	1	2
173	−	−	−	−			+	+			138	1.28	2	1
129	−	−					+	+			141	1.15	1	1
149						+	+			+	>145	0.05	0	3
177						+	+	+			129	1.20	0	2
Total	5	6	7	7	2/0	5	7	9	2	6	135.3	0.53	1.21	1.57

*PA* phonological awareness, *RAN* rapid automatized naming, *VSTM* verbal short-term memory, *VWM* verbal working memory, *VSSTM* visuospatial short-term memory, *VSWM* visuospatial working memory, *SS* standard score

The case series analysis shows that both groups contain about equal percentages of children with a deficit in PA (i.e., gifted/dyslexic (GD) = 46.2 %, borderline (B) = 50.0 %) and the gifted/dyslexic group has a higher percentage of children with a RAN deficit than the borderline group (i.e., GD = 76.9 %, B = 57.1 %). In other words, despite the more severe PA deficit for the gifted/dyslexic group compared to the borderline group, the gifted/dyslexic group did not include *more* children with a deficit. Only 7.7 % of the gifted/dyslexic children and 14.3 % of the borderline children show a deficit in VSTM. On the other hand, none of the borderline children and only 15.3 % of the gifted children with dyslexia show a strength in VSTM.

Besides individual deficits, combinations of deficits can play a role; the gifted/dyslexic group might show accumulation of risk factors for the development of severe literacy difficulties. Tables 5 and 6 show that both groups have about the same number of weaknesses on average. Although the groups are comparable in their percentages of children with no (GD = 15.4 %, B = 14.3 %) or three deficits (GD = 7.7 %, B = 7.1 %), the borderline group contains more children with a single deficit (GD = 46.2 %, B = 57.1 %) and the gifted/dyslexic group more children with a double deficit (i.e., mostly a combination between PA and RAN; GD = 30.8 %, B = 21.4 %). This higher percentage of children with a double deficit in the gifted/dyslexic group directly translates to the higher percentage of children with a RAN deficit in this group mentioned above.

The case series analysis on the *protective* factors largely confirms the pattern that the group comparison of the gifted children with dyslexia and the borderline children showed. The gifted/dyslexic group contains a lower percentage of children with a strength in verbal WM (i.e., GD = 23.1 %, B = 35.7 %). Furthermore, in both groups, half of the children show a strength in language skills, including grammar, of which the group comparisons indicated that it might not be a strength in gifted/dyslexic children overall. Remarkably, visual WM is a strength for many of the children in the gifted/dyslexic group, even compared to the borderline group (i.e., GD = 92.3 %, B = 71.4 %). These percentages show that the borderline children do not have more or more relevant protective factors that may explain their higher literacy levels, which is supported by the percentages of combinations of strengths. Both groups have about equal percentages of children with two (i.e., GD = 38.5 %, B = 42.9 %) or three strengths (i.e., GD = 15.4 %, B = 14.3 %). However, the gifted/dyslexic group contains fewer children with no strengths at all (i.e., GD = 3.8 %, B = 14.3 %) and more children with a single strength (i.e., GD = 42.3 %, B = 28.6 %). This higher percentage of children with a single strength is directly related to the higher number of children with a strength in visual WM in the gifted/dyslexic group.

## Discussion

In the present study, we aimed to test and extend theories on cognitive risk and protective factors in dyslexia, by investigating cognitive profiles of gifted children at the group and individual levels. In order to gain more insight in possibilities for compensation and masking of learning disabilities, three groups were compared in the light of two competing views; the core-deficit view (e.g., Stanovich, 1996) and the twice-exceptionality view (e.g., Assouline et al., 2010). First, a comparison was made between gifted children with dyslexia and averagely intelligent children with dyslexia, controlling for literacy level. Second, gifted children with dyslexia and borderline-dyslexic children (i.e., gifted children with relative literacy problems) were compared, controlling for differences in overall intelligence. Third, individual profiles within both gifted groups were investigated to provide more information about differences in overall numbers of risk and protective factors, the breadth/depth of underlying deficits, and the specificity of strengths.

The first comparison showed that gifted children with dyslexia had similar (low) scores on RAN compared to averagely intelligent children, but also seemingly less severe deficits in PA and VSTM. Evidently, gifted children with dyslexia do not have larger underlying deficits than children with dyslexia, indicating no support for the twice-exceptionality view. The similar scores on RAN in both groups provide partial support for the core-deficit view. Moreover, the gifted children with dyslexia showed high performance across a broad range of protective factors. The equal scores on grammar, which is assumed to be more strongly related to literacy than most of the other factors, suggests that the high scores on the other protective factors are relatively independent of the scores on the risk factors and the literacy performance. This is also largely in line with the core-deficit view. The finding that RAN levels of gifted children and averagely intelligent children with dyslexia were similar indicates that a RAN deficit is an important risk factor underlying the literacy deficits in both groups.

The second comparison indicates that the higher literacy levels of the borderline children compared to those of the gifted children with dyslexia could be explained by less severe underlying cognitive deficits of the borderline group, in line with the core-deficit view. Although the multivariate results indicated that the profiles of risk factors were comparable between both groups, the univariate results showed that gifted children with dyslexia on average have more severe deficits in PA and RAN. In addition, the case series analyses indicated that the phonological problems of the gifted children with dyslexia not only are on average more severe but also more often tend to concern a combination of deficits than in the borderline children. Both groups had about equal scores on VSTM. Coupling the VSTM findings to the low percentage of children with a deficit in the case series analysis, VSTM does not seem to be a general weakness in all gifted children with literacy difficulties. This is in line with previous findings by van Viersen et al. (2014) as well as findings in the general population (Moll, Loff, & Snowling, 2013). However, the low percentage of children with above average scores on VSTM shows that it might not be a strength either and illustrates the relatively strong performance of gifted children on this task compared to averagely intelligent children with dyslexia.

The hypothesis that borderline children might have higher literacy levels because they possess specific strengths more relevant to literacy development (e.g., language skills), as suggested by the twice-exceptionality view, was not supported. In line with their equal intelligence levels, borderline children and gifted children with dyslexia largely share their profile of cognitive strengths and showed comparable percentages of children with a strength in language skills, reducing the possibility of domain-specific compensation. The borderline children only outperformed gifted children with dyslexia on verbal WM, both in group means as well as number of borderline children with a strength in this area. Verbal WM may not be a strength in gifted children with dyslexia because it is related to phonological memory (Vellutino et al., 2004), which is more impaired in gifted children with dyslexia than in the borderline children. On the other hand, for almost all gifted children with dyslexia, visual WM was considered a strength. Apparently, there are relatively more children in the gifted/dyslexic group than in the borderline group who have a single strength in visual WM, but broader impairments in phonology and language-related skills, which provides an indication of the breadth and specificity of the problems of these children.

Based on these findings, it can be stated that, in line with the core-deficit view, literacy performance (measured by core tasks generally used in diagnosing dyslexia) and the associated underlying cognitive deficits seem to be largely independent of intelligence (Stanovich, 1996), *also* in gifted populations with literacy problems. The counterevidence, i.e., the higher scores on PA and VSTM of the gifted children with dyslexia compared to the averagely intelligent children with dyslexia, may (partly) be the result of the “task impurity problem” (van der Sluis, de Jong, & van der Leij, 2007), which concerns compensation on task-related aspects. Skills involved in on-task compensation may either be related to literacy ability, such as a larger vocabulary leading to higher familiarity with words in the task, or unrelated to literacy ability, i.e., higher processing speed (Catts, Gillispie, Leonard, Kail, & Miller, 2002; Johnson, Im-Bolter, & Pascual-Leone, 2003). For example, both PA tasks were timed and “phonological speed” of the children played a prominent role in the assessment of task performance. This possibility is supported by the findings of the comparison between both gifted groups. The borderline children showed less severe deficits and no specific strengths, explaining their higher literacy levels, while resources for compensation on task-related aspects were equal across both groups.

As stated earlier, most studies appear to agree that compensation pertains to the moderation of the effect of a cognitive risk factor by an additional or co-occurring protective factor, but it remains unclear how this might actually work. The current study found no evidence for compensation by IQ-related factors as proposed by the twice-exceptionality view. Accordingly, compensation does not entail a balance between risk and protective factors (at a specific point in time), resulting in remediation of the defective underlying skill and better literacy performance at the outcome level. However, other types of compensation remain relevant in relation to gifted children. For example, even though the task impurity problem involves compensation on task-specific aspects and not compensation in relation to a defective skill, it is clear that this type of compensation poses serious challenges to diagnostic practice. The higher task performance ensuing from task-related compensation frustrates finding and proving the existence of a severe underlying deficit, providing an incorrect picture of the child’s skills and deficits.

Another interesting option, next to on-task compensation, is compensation by a protective factor *during* development, resulting in the emergence of compensatory mechanisms or strategies that can be used in more complex literacy tasks, such as text reading and reading comprehension (e.g., Nation & Snowling, 1998). This type of compensation implies that there may be alternative ways to cope with problems that are involved in literacy performance, resulting in better performance at the outcome level than would be predicted based on the deficient underlying skill. This type of compensation is also explicitly proposed within the twice-exceptional framework (e.g., Reis, McGuire, & Neu, 2000). Possible factors involved in this type of compensation might be the amount of print exposure (Mol & Bus, 2011) and orthographic learning (Wang, Castles, Nickels, & Nation, 2011). For example, when gifted children with dyslexia are better able to learn orthographic knowledge (i.e., letter clusters) than averagely intelligent children with dyslexia, they read and recognize longer words and/or words of larger complexity (Bekebrede, Van der Leij, & Share, 2009). This orthographic knowledge can be used as a compensatory mechanism to *circumvent* their phonological deficit and improve their performance on the literacy task (van der Leij & van Daal, 1999). This type of compensation is more in line with literacy outcomes determined by the multiple deficit model, because it underlines the developmental nature of interactions between risk and protective factors and illustrates why protective factors should be taken into account in theories on dyslexia.

The present results suggest that borderline children do not compensate underlying deficits, masking their literacy difficulties to an extent that they unjustly miss a dyslexia diagnosis. The term “borderline-dyslexic” is thus appropriate, as the children’s literacy problems may not be severe enough to qualify for dyslexia. Yet, borderline children experience educational problems directly related to the *consequences* of the discrepancy between their high intelligence and lower literacy levels. Elbro (2010) refers to this issue by differentiating between dyslexia as a disability, i.e., poor ability, and dyslexia as a handicap, i.e., the consequences of poor ability. For these children, their handicap may predominantly lie in the discrepancy between their literacy level and the difficulty of the texts they are supposed to be able to read based on their intelligence.

The results do provide implications for diagnostic practice. Given that the relation between phonological deficits and literacy difficulties is not causal (e.g., Pennington, 2006), we contend that it is important to continue to diagnose dyslexia based on literacy performance at the behavioral level and *not* include literacy-related cognitive deficits as a requirement for a diagnosis. Mapping of risk and protective factors should solely be used for underpinning a diagnosis and or providing clues for intervention. With respect to the borderline children, adhering to diagnosis at the behavioral level would confirm that these children are indeed not dyslexic based on their literacy levels. However, future research should establish whether these children might actually experience educational problems as the result of their relative literacy problems or “handicap” (e.g., Assouline, Foley Nicpon, & Huber, 2006; Elbro, 2010), especially since the decision as to whether a child is eligible for remediation or intervention is dependent on the diagnosis of dyslexia.

There are some limitations to this study. First, due to the proximal analysis of literacy and underlying cognitive abilities, it was not possible to *directly* test mechanisms of compensation and masking in the present study. Instead, inferences were based on patterns of literacy and cognitive skills across groups and individual children. More information is needed about the way skills included here contribute to literacy processes to make more detailed statements about compensation and masking in future studies. Furthermore, our sample size was relatively small, including a limited group of borderline children, and the effects attested were not very large. Our choice for a Bayesian approach instead of traditional statistics allowed for the comparison of two opposing views and effectively dealt with the problems associated with small groups that are generally found at the end of the normal distribution. Combined with a detailed case series analyses, it allowed us to visualize the patterns of risk and protective factors and showed a clear trend toward distinctive cognitive characteristics in both gifted groups. Nonetheless, the results should be interpreted with caution and warrant replication in larger samples and different age groups.

In summary, this study confirmed that the profiles of underlying cognitive risk factors of children with dyslexia are largely independent of intelligence, also in gifted populations. We found no clear evidence for direct compensation of dyslexia-related cognitive risk factors by giftedness-related (and literacy-related) protective factors in gifted children with dyslexia. Moreover, gifted children with dyslexia and borderline-dyslexic children can be distinguished based on their literacy levels and the severity and breadth/depth of their underlying cognitive deficits. There was no evidence for compensation through specific protective factors that would further explain the differences in literacy levels between both gifted groups. Yet, other types of compensation remain possible, illustrating the importance of further investigating the role of protective factors and extend theories as well as aid diagnostic practice.

## Electronic supplementary material

Online Resource 1 (DOCX 22.2 kb)

## Footnotes

1 Please note that, although the differences in profiles between the gifted/dyslexic group and the borderline group do not seem to be large, both gifted groups significantly differ from averagely intelligent children with dyslexia in their number of risk and protective factors as well as the breadth/depth of their weaknesses and strengths. Summary of the results for the case series analysis of the averagely intelligent children with dyslexia: PA deficit = 87.9 %, RAN deficit = 84.8 %, VSTM deficit = 39.3 %; no deficits = 0.0 %, one deficit = 18.2 %, two deficits = 51.5 %, three deficits = 30.3 %; verbal WM strength = 0.0 %, visual WM strength = 27.3 %, language strength = 12.1 %; no strengths = 60.6 %, one strength = 39.4 %, two strengths = 0.0 %, three strengths = 0.0 %. The complete table is displayed in Online Resource 1.

## References

[CR1] Alloway TP (2007). Automated Working Memory Assessment (AWMA).

[CR2] Alloway TP, Gathercole SE, Kirkwood H, Elliot J (2009). The cognitive and behavioral characteristics of children with low working memory. Child Development.

[CR3] Assouline SG, Foley Nicpon M, Huber DH (2006). The impact of vulnerabilities and strengths on the academic experiences of twice-exceptional students: A message to school counselors. Professional School Counseling.

[CR4] Assouline SG, Foley Nicpon M, Whiteman C (2010). Cognitive and psychosocial characteristics of gifted students with written language disability. Gifted Child Quarterly.

[CR5] Bekebrede JI, Van der Leij A, Share DL (2009). Dutch dyslexic adolescents: Phonological-core variable-orthographic differences. Reading and Writing: An Interdisciplinary Journal.

[CR6] Berninger VW, Abbott RD (2013). Differences between children with dyslexia who are and are not gifted in verbal reasoning. Gifted Child Quarterly.

[CR7] Bishop DVM, Snowling MJ (2004). Developmental dyslexia and specific language impairment: Same or different?. Psychological Bulletin.

[CR8] Boets B, de Smedt B, Cleuren L, Vandewalle E, Wouters J, Ghesquière P (2010). Towards a further characterization of phonological and literacy problems in Dutch speaking children with dyslexia. British Journal of Developmental Psychology.

[CR9] Brody LE, Mills CJ (1997). Gifted children with learning disabilities: A review of the issues. Journal of Learning Disabilities.

[CR10] Brus BT, Voeten MJM (1999). Eén-minuut-test [One-minute-test].

[CR11] Catts HW, Gillispie M, Leonard LB, Kail RV, Miller CA (2002). The role of speed of processing, rapid naming, and phonological awareness in reading achievement. Journal of Learning Disabilities.

[CR12] Catts HW, Hogan TP, Fey M (2003). Subgrouping poor readers on the basis of reading-related abilities. Journal of Learning Disabilities.

[CR13] de Jong PF, van der Leij A (2003). Developmental changes in the manifestation of a phonological deficit in dyslexic children learning to read a regular orthography. Journal of Educational Psychology.

[CR14] Elbro C (2010). Dyslexia as disability or handicap: When does vocabulary matter?. Journal of Learning Disabilities.

[CR15] Evers, A., Egberink, I. J. L., Braak, M. S. L., Frima, R. M., Vermeulen, C. S. M., & Van Vliet-Mulder, J. C. (2009–2012). *COTAN documentatie [COTAN documentation]*. Amsterdam: Boom testuitgevers.

[CR16] Foley Nicpon M, Allmon A, Sieck B, Stinson RD (2011). Empirical investigation of twice-exceptionality: Where have we been and where are we going?. Gifted Child Quarterly.

[CR17] Geelhoed J, Reitsma P (2000). PI-dictee [PI-dictation].

[CR18] Gill J (2008). Bayesian methods: A social and behavioral sciences approach.

[CR19] Johnson J, Im-Bolter N, Pascual-Leone J (2003). Development of mental attention in gifted and mainstream children: The role of mental capacity, inhibition, and speed of processing. Child Development.

[CR20] Kass RE, Raftery AE (1995). Bayes factors. Journal of the American Statistical Association.

[CR21] Kaufman AS, Kaufman JC, Balgopal R, McLean JE (1996). Comparison of three WISC-III short forms: Weighing psychometric, clinical, and practical factors. Journal of Clinical Child Psychology.

[CR22] Kleijnen, R., Bosman, A., de Jong, P., Henneman, K., Pasman, J., Paternotte, A., … Wijnen, F. (2008). *Diagnose en Behandeling van Dyslexie. Brochure van de Stichting Dyslexie Nederland [Diagnosis and treatment of dyslexia. Brochure of the Dutch Dyslexia Foundation]*. Bilthoven: Stichting Dyslexie Nederland.

[CR23] Klugkist I, Laudy O, Hoijtink H (2005). Inequality constrained analysis of variance: A Bayesian approach. Psychological Methods.

[CR24] Klugkist I, van Wesel F, Bullens J (2011). Do we know what we test and do we test what we want to know?. International Journal of Behavioral Development.

[CR25] Kort, W., Schittekatte, M., Bosmans, M., Compaan, E. L., Dekker, P. H., Vermeir, G., & Verhaege, P. (2005). *WISC III-NL. Wechsler Intelligence Scale for Children III-NL.* Amsterdam, The Netherlands: Pearson.

[CR26] Kort W, Schittekatte M, Compaan EL (2010). CELF-4-NL. Clinical Evaluation of Language Fundamentals-4 NL.

[CR27] Lovett BJ, Lewandowski LJ (2006). Gifted students with learning disabilities: Who are they?. Journal of Learning Disabilities.

[CR28] Mol SE, Bus AG (2011). To read or not to read: A meta-analysis of print exposure from infancy to early adulthood. Psychological Bulletin.

[CR29] Moll K, Loff A, Snowling MJ (2013). Cognitive endophenotypes of dyslexia. Scientific Studies of Reading.

[CR30] Mulder J, Hoijtink H, de Leeuw C (2012). BIEMS: A fortran 90 program for calculating Bayes factor for inequality and equality constrained models. Journal of Statistical Software.

[CR31] Mulder J, Hoijtink H, Klugkist I (2010). Equality and inequality constrained multivariate linear models: Objective model selection using constrained posterior priors. Journal of Statistical Planning and Inference.

[CR32] Mulder J, Klugkist I, Meeus W, van de Schoot A, Selfhout M, Hoijtink H (2009). Bayesian model selection of informative hypotheses for repeated measurements. Journal of Mathematical Psychology.

[CR33] Nag S, Snowling MJ (2011). Cognitive profiles of poor readers of Kannada. Reading and Writing.

[CR34] Nation K, Snowling MJ (1998). Individual differences in contextual facilitation: Evidence from dyslexia and poor reading comprehension. Child Development.

[CR35] Pennington BF (2006). From single to multiple deficit models of developmental disorders. Cognition.

[CR36] Pennington, B. F., Santerre-Lemmon, L., Rosenberg, J., MacDonald, B., Leopold, D. R., Byrne, B., … & Olson, R. K. (2012). Individual prediction of dyslexia by single versus multiple deficit models. *Journal of Abnormal Psychology, 121*, 212–224. doi:10.1037/a0025823 10.1037/a0025823 PMC3270218 22022952

[CR37] Ramus, F., Rosen, S., Dakin, S. C., Day, B. L., Castellote, J. M., White, S., & Frith, U. (2003). Theories of developmental dyslexia: Insights from a multiple case study of dyslexic adults. *Brain, 126*, 841–865. doi:10.1093/brain/awg076 10.1093/brain/awg076 12615643

[CR38] Reis SM, McGuire JM, Neu TW (2000). Compensation strategies used by high ability students with learning disabilities who succeed in college. Gifted Child Quarterly.

[CR39] Silverman LK, Colangelo N, Davis G (2003). Gifted children with learning disabilities. Handbook of gifted education.

[CR40] Snowling MJ (1998). Dyslexia as a phonological deficit: Evidence and implications. Child Psychology & Psychiatry Review.

[CR41] Snowling MJ (2000). Dyslexia.

[CR42] Snowling, M. J. (2001). From language to reading and dyslexia. *Dyslexia, 7*, 37–46. doi:10.1002:dys.185. 10.1002/dys.185 11305230

[CR43] Snowling MJ (2008). Specific disorders and broader phenotypes: The case of dyslexia. The Quarterly Journal of Experimental Psychology.

[CR44] Snowling MJ, Gallagher A, Frith U (2003). Family risk of dyslexia is continuous: Individual differences in the precursors of reading skill. Child Development.

[CR45] Stanovich KE (1996). Toward a more inclusive definition of dyslexia. Dyslexia.

[CR46] Stanovich KE, Siegel LS (1994). Phenotypic performance profile of children with reading disabilities: A regression-based test of the phonological-core variable difference model. Journal of Educational Psychology.

[CR47] van den Bos KP, Lutje Spelberg HC (2007). Continu Benoemen & Woorden Lezen (CB&WL) [Continuous naming & word reading].

[CR48] van den Bos KP, lutje Spelberg HC, Scheepstra AJM, de Vries JR (1994). De Klepel. Vorm A en B [Nonword reading test].

[CR49] van den Bos KP, lutje Spelberg HC, de Groot BJA (2011). Fonemische Analyse Test (FAT) [Phonemic analysis test].

[CR50] van der Leij A, van Daal VHP (1999). Automatization aspects of dyslexia: Speed limitations in word identification, sensitivity to increasing task demands, and orthographic compensation. Journal of Learning Disabilities.

[CR51] van der Sluis S, de Jong PF, van der Leij A (2007). Executive functioning in children, and its relations with reasoning, reading, and arithmetic. Intelligence.

[CR52] van Viersen S, Kroesbergen EH, Slot EM, de Bree EH (2014). High reading skills mask dyslexia in gifted children. Journal of Learning Disabilities.

[CR53] Vellutino FR, Fletcher JM, Snowling MJ, Scanlon DM (2004). Specific reading disability (dyslexia): What have we learned in the past four decades?. Journal of Child Psychology and Psychiatry.

[CR54] Vellutino FR, Scanlon DM, Lyon GR (2000). Differentiation between difficult-to remediate and readily remediated poor readers: More evidence against the IQ achievement discrepancy definition of reading disability. Journal of Learning Disabilities.

[CR55] Wagner RK, Torgesen JK (1987). The nature of phonological processing and its causal role in the acquisition of reading skills. Psychological Bulletin.

[CR56] Wang H-C, Castles A, Nickels L, Nation K (2011). Context effects on orthographic learning of regular and irregular words. Journal of Experimental Child Psychology.

[CR57] Winner E (1997). Exceptionally high intelligence and schooling. American Psychologist.

[CR58] Wolf M, Bowers PG (1999). The double-deficit hypothesis for the developmental dyslexias. Journal of Educational Psychology.

