# Supplementary material for: Risk and protective factors in gifted children with dyslexia
Source: Ann Dyslexia. 2015 Aug 13;65(3):178–98. doi: 10.1007/s11881-015-0106-y (PMC4565890; doi:10.1007/s11881-015-0106-y)
Supplement: Supplementary file 1 — (DOCX 22.2 kb) [file 11881_2015_106_MOESM1_ESM.docx]

**Online Resource 1**

Risk and Protective Factors in Gifted Children with Dyslexia

Sietske van Viersen^1,2^*, Elise H. de Bree^1^, Evelyn H. Kroesbergen^2^, Esther M. Slot^2^, & Peter F. de Jong^1^

*Corresponding author (S.vanViersen@uva.nl, Phone: +31 (0)20 525 7839)

^1^University of Amsterdam
Research Institute of Child Development and Education (RICDE)

PO Box 15780

1001 NG Amsterdam

The Netherlands

^2^Utrecht University

Department of Pedagogical and Educational Sciences

PO Box 80140

3508 TC Utrecht

The Netherlands

Table A

*Case-Series showing the Cognitive Risk and Protective Factors observed among Averagely Intelligent Children with Dyslexia*

|  | Riskfactors | | | | | Protective factors | | | | |  | | |
| --- | --- | --- | --- | --- | --- | --- | --- | --- | --- | --- | --- | --- | --- |
| Case no. | PA deletion  SS < 8 | PA  spoonerism  SS < 8 | RAN alpha-numeric  SS < 8 | RAN non-alphanumeric  SS < 8 | VSTM  SS < 85  SS > 115 | VWM  SS > 115 | VSSTM  SS > 115 | VSWM  SS > 115 | Grammar  SS > 12 | Voca-bulary  SS > 12 | IQ-score | Number of weaknesses | Number of strengths |
| 76 | - | - | - | - |  |  | + | - |  | - | 91 | 2 | 1 |
| 153 |  |  | - | - |  |  |  |  | + |  | 111 | 1 | 1 |
| 207 |  |  | - | - |  |  | + | + |  |  | 112 | 1 | 1 |
| 95 | - |  | - | - |  | - | - | - |  |  | 84 | 2 | 0 |
| 189 | - |  |  | - | - |  | - | - |  |  | 87 | 3 | 0 |
| 201 | - |  | - | - | - | - |  | - |  | - | 88 | 3 | 0 |
| 116 | - |  |  |  |  |  |  | + |  |  | 110 | 1 | 1 |
| 77 | - |  | - | - | - | - |  |  |  | - | 90 | 3 | 0 |
| 188 | - | - | - |  | - | - |  |  |  |  | 89 | 3 | 0 |
| 119 | - | - |  |  | - | - | - | - |  | - | 100 | 2 | 0 |
| 123 | - |  | - | - | - | - | - | - |  | - | 92 | 3 | 0 |
| 190 |  |  | - | - |  |  |  | - |  | - | 89 | 1 | 0 |
| 117 | - |  | - | - |  |  |  | - |  |  | 100 | 2 | 0 |
| 72 | - | - | - | - |  |  |  | - |  | - | 108 | 2 | 0 |
| 64 | - | - |  |  | - |  | + |  |  | - | 111 | 2 | 1 |
| 70 | - | - | - | - | - |  | + | + |  | - | 102 | 3 | 1 |
| 21 | - |  | - | - |  |  |  |  | + |  | 118 | 2 | 1 |
| 89 | - | - | - | - |  | - | - | - | + |  | 98 | 2 | 1 |
| 215 | - | - | - | - |  |  |  |  |  |  | 95 | 2 | 0 |
| 135 | - | - | - | - |  |  | + | - |  | - | 104 | 2 | 1 |
| 137 | - | - | - | - |  |  |  | - |  | - | 71 | 2 | 0 |
| 209 | - | - | - | - |  |  | + | + |  |  | 121 | 2 | 1 |
| 88 | - |  | - |  |  |  |  |  |  |  | 92 | 2 | 0 |
| 127 | - | - | - | - | - |  |  |  |  | - | 104 | 3 | 0 |
| 214 | - | - | - | - |  |  | + | + |  |  | 105 | 2 | 1 |
| 71 | - | - | - |  |  |  |  |  |  |  | 103 | 2 | 0 |
| 231 | - | - | - | - | - |  |  | - |  | - | 86 | 3 | 0 |
| 232 | - | - | - |  |  |  |  | - |  |  | 86 | 2 | 0 |
| 96 | - |  | - | - | - | - |  | - |  |  | 102 | 3 | 0 |
| 147 |  | - |  |  |  |  |  | - |  |  | 97 | 1 | 0 |
| 172 | - | - | - | - | - | - | - | - |  | - | 95 | 3 | 0 |
| 218 |  |  | - | - | - |  |  | - | + | - | 96 | 2 | 1 |
| 47 | - | - |  |  |  |  | + | + |  |  | 120 | 1 | 1 |
| Total | 28 | 19 | 27 | 24 | 13 | 0 | 8 | 6 | 4 | 0 | 98.70 | 2.12 | 0.40 |

*Note*. PA = phonological awareness, RAN = rapid automatized naming, VSTM = verbal short-term memory, VWM = verbal working memory, VSSTM = visuospatial short-term memory, VSWM = visuospatial working memory, SS = standard score.
